# Proposals Generation for Weakly Supervised Object Detection in Artwork Images

**DOI:** 10.3390/jimaging8080215

**Published:** 2022-08-06

**Authors:** Federico Milani, Nicolò Oreste Pinciroli Vago, Piero Fraternali

**Affiliations:** Department of Electronics Information and Bioengineering, Politecnico di Milano, 20133 Milano, Italy

**Keywords:** weakly supervised learning, wsod, class activation maps, artworks, cultural heritage

## Abstract

Object Detection requires many precise annotations, which are available for natural images but not for many non-natural data sets such as artworks data sets. A solution is using Weakly Supervised Object Detection (WSOD) techniques that learn accurate object localization from image-level labels. Studies have demonstrated that state-of-the-art end-to-end architectures may not be suitable for domains in which images or classes sensibly differ from those used to pre-train networks. This paper presents a novel two-stage Weakly Supervised Object Detection approach for obtaining accurate bounding boxes on non-natural data sets. The proposed method exploits existing classification knowledge to generate pseudo-ground truth bounding boxes from Class Activation Maps (CAMs). The automatically generated annotations are used to train a robust Faster R-CNN object detector. Quantitative and qualitative analysis shows that bounding boxes generated from CAMs can compensate for the lack of manually annotated ground truth (GT) and that an object detector, trained with such pseudo-GT, surpasses end-to-end WSOD state-of-the-art methods on ArtDL 2.0 (≈41.5% mAP) and IconArt (≈17% mAP), two artworks data sets. The proposed solution is a step towards the computer-aided study of non-natural images and opens the way to more advanced tasks, e.g., automatic artwork image captioning for digital archive applications.

## 1. Introduction

Object Detection (OD) aims at identifying the location of objects inside an image and plays an important role in many applications: surveillance [1], automotive [2], medical imaging [3], remote sensing [4] and figurative art [5]. Object detection in figurative art supports iconology, which aims at tracking the spatio-temporal diffusion of symbols (objects) across artworks to study the influences and propagation of ideas. For example, in crucifixion scenes, the presence and number of nails and ropes helps date the artwork, the upper branch of the cross helps determine the geographic provenance, and in Mary Magdalene images, a book is a hint to the influence of Gnosticism. Since large digital artwork archives are spreading, computer-aided iconology at scale may support the creation of new knowledge.

However, training a fully supervised object detector requires a large number of precisely annotated bounding boxes. Such annotations may be challenging to acquire in novel domains, e.g., cultural heritage, due to the effort of the annotation task. Moreover, large-scale crowd-sourcing campaigns, e.g., Amazon Mechanical Turk, may lead to inaccurate and inconsistent results in domains that require expert knowledge. Weakly Supervised Object Detection (WSOD) aims to learn to localize classes inside an image using image-level annotations only.

Initially, WSOD methods addressed the task with Multiple Instance Learning (MIL) by finding high-confidence region proposals based on positive image-level annotations [6,7,8]. More recent works employ the Weakly Supervised Deep Detection Network (WSDDN), which combines MIL and Convolutional Neural Networks (CNNs) and enhance the architecture with specific modules to improve instance classification and localization [9]. State-of-the-art studies have demonstrated outstanding results on natural images, but three main problems arise when dealing with non-natural images. First, the methods rely on external proposals, generated with Selective Search [10] or Region Proposal Networks (RPNs) [11], which are difficult to produce when the target data set does not share classes with the pre-trained RPNs models. Secondly, the complexity and noisiness of non-natural data sets have a negative impact on the learning process [12]. Common problems are confusing backgrounds, the density of instances, less discriminative class features, large intra-class diversity and inter-class similarity, artificial colours and shadows, and low image quality. Finally, most state-of-the-art end-to-end WSOD methods freeze a large part of the network, thus making it impossible to take advantage of the renowned benefits of Transfer Learning (TL) on non-natural data sets [13,14,15].

**Figure 1:**
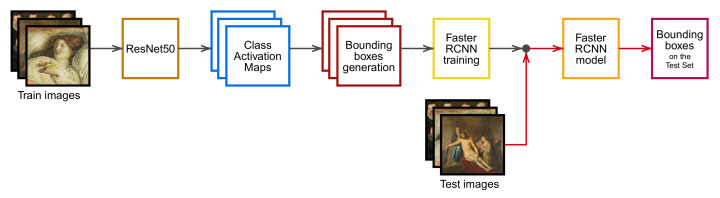
**Proposed pipeline**—The pipeline comprises several steps: (1) training of a ResNet-50 classifier on train set images; (2) computation of Class Activation Maps; (3) devising of bounding boxes surrounding the connected components on train set images; (4) use of the obtained bounding boxes as pseudo-GT to train a Faster R-CNN object detector; and (5) application of the trained detector model on test images. Gray arrows indicate the training steps, while red arrows indicate the testing steps.

For these reasons, this paper presents a training pipeline (Figure 1) composed of three stages: a ResNet-50 classifier, a Class Activation Map (CAM) technique and a Faster R-CNN object detector. The resulting architecture fully exploits the strength of TL when training the classifier and the object detector, the visualization capabilities of CAMs and the robustness and efficiency of Faster R-CNN.

The contributions can be summarized as follows:

- We propose a WSOD approach based on three components that can be fully customized to work on non-natural data sets where state-of-the-art architectures fail. The proposed pipeline consists of: an existing ResNet-50 classifier, a CAM method paired with a dynamic thresholding technique to generate pseudo-ground truth (pseudo-GT) bounding boxes, and a Faster R-CNN object detector to localize classes inside images.
- We evaluate performances on two artwork data sets (ArtDL 2.0 and IconArt [16]), which are annotated for WSOD and whose complexity has been demonstrated in previous studies [17,18]. Our approach is able to reach ≈41.5% mAP on ArtDL and ≈17% mAP on IconArt, where state-of-the-art techniques obtain a maximum value of ≈25% and ≈15% mAP, respectively.
- We provide qualitative analysis to highlight the ability of the object detector to correctly localize multiple classes/instances even in complex artwork scenes. While the object detector can uncover features that are not found by the original classifier, failure examples show that the model sometimes suffers the inaccuracy of the pseudo-GT annotations used for training.
- For our analysis, we have extended an existing data set (ArtDL [14]) with 1697 manually annotated bounding boxes on 1625 images.

The rest of the paper is organized as follows: Section 2 surveys related work; Section 3 describes the proposed pipeline; Section 4 evaluates each stage of the proposed pipeline quantitatively and qualitatively and compares the performance with state-of-the-art techniques; finally, Section 5 draws the conclusions and outlines the future work.

## 2. Related work

This section surveys the state-of-the-art WSOD algorithms and computer vision applications on artwork images.

### 2.1. Weakly Supervised Object Detection

WSOD methods aim to localize classes inside an image using limited annotations, usually image-level labels. Initially, the task has been tackled as a MIL problem [6,7,8] in which images are treated as bags of positive or negative instances for each class based on the image-level annotations. A classifier is trained to distinguish each generated proposal’s most discriminative features and assign a category to them. MIL represents a non-convex optimization problem, thus the training may get stuck in a local minimum. Several studies propose a solution either by constraining the initialization of the network [19,20] or by modifying the learning process [21,22]. Interesting approaches are presented by [21,23] and [22], which optimize the problem by dividing it into sub-parts, respectively, at the data level and loss level. Recent works combine MIL and Deep Neural Networks (DNN) in WSDDNs [9,24,25,26,27]. A typical WSDDN is composed of two streams, devoted to classification and localization trained jointly to mine positive samples [28]. Several studies build upon WSDDN and try to refine the proposal localization: OICR [29] introduces multiple online instance classifiers to select more accurate boxes and PCL [30] clusters proposals based on similar image features and uses them for training supervision. The authors of [31,32,33,34,35] integrate single or multiple bounding boxes regressors into their architectures to perform re-localization. Since all these methods require external proposals, UWSOD [36] proposes a unified framework that exploits self-supervision for bounding box proposal and refinement. All the cited methods are still limited by the MIL non-convex optimization problem. Many studies combine CAMs [37] or Weakly Supervised Semantic Segmentation (WSSS) [38] to achieve better WSOD performances. The authors of [39,40,41] leverage the power of CAMs as segmentation proposals,  [42,43,44,45] introduce a collaboration loop between the segmentation and detection branches,  [46] proposes a cascaded convolutional neural network and [47] exploit segmentation properties, i.e., purity and completeness, to harvest tight boxes that take into account the surrounding context. Still, the actual methods cannot fully exploit CAMs as bounding box generators and require the use of external domain-dependent proposals or hybrid-annotated data. In addition, many architectures use erasing techniques, which have been proven to be detrimental, especially when multiple similar classes appear in the same image [48]. The authors of [49] survey MIL-based and CAM-based approaches for WSOD, presenting quantitative results on four benchmarking data sets.

### 2.2. Automated Artwork Image Analysis

In recent years, there has been an increasing interest in applying Artificial Intelligence in the cultural heritage field [5,50]. Studies have been supported by the massive digitization of artworks and the release of public data sets. Still, most studies focus on style and material recognition [51,52] or author classification [53], while few tackle visual question answering [54,55] or captioning [56,57]. Object Detection is still a rarely studied task in the cultural heritage field [58,59,60], mainly due to the scarcity of large-scale annotated data sets [61] and the low similarity with natural image classes. Annotating artworks requires previous knowledge and is unsuitable for public crowd-sourcing campaigns, especially when dealing with highly specific classes, e.g., Iconclasses [62], and scenes characterized by multiple subjects scattered in the entire image. Image-level labels are way more straightforward and less time-consuming to annotate; they can often be retrieved by descriptions or properties in the digital collection (e.g., title, author, date, depicted classes, etc.). Very few works leverage weak annotations for WSOD on artworks. [63] is a pioneering work on the task and proposes a weakly-supervised approach for localizing gods and animals on Greek vases. The authors of [16,18] present a novel data set, IconArt, and a MIL classifier trained on proposals obtained by Faster R-CNN. The research in  [17] studies the efficacy of Class Activation Maps in localizing relevant iconography symbols of Christian Iconography on the ArtDL [14] data set. [64] introduces a cross-domain WSOD framework to localize objects in watercolour images. Domain adaptation is obtained through the style transfer of fully-annotated natural images. The technique is limited to classes that exist and that have been annotated in other domains. A key challenge of object identification and localization in the cultural heritage domain is the adverse training environment given by low distinctiveness of class features, high heterogeneity of class representations, confusing backgrounds, and image quality. These conditions require the study of specific techniques and network adaptations or combinations.

## 3. Methods

This section introduces the proposed weakly supervised object detection pipeline shown in Figure 1. Once the ResNet-50 classification architecture has been trained with image-level labels, weak bounding boxes are extracted from CAMs and exploited as pseudo-GT to train a Faster R-CNN object detector. Finally, test set bounding boxes are obtained by feeding images directly to the object detection model without computing CAMs on the test set.

### 3.1. Classification Architecture

The first stage of the pipeline shown in Figure 1 consists in training a fully supervised classification model with image-level labels. We employ ResNet-50 [65], a well-known CNN architecture, pre-trained on the ImageNet data set [66]. The network was chosen for the outstanding classification results obtained even on non-natural images, thanks to transfer learning [14,52,67,68].

#### Discriminative Region Suppression

**Figure 2:**
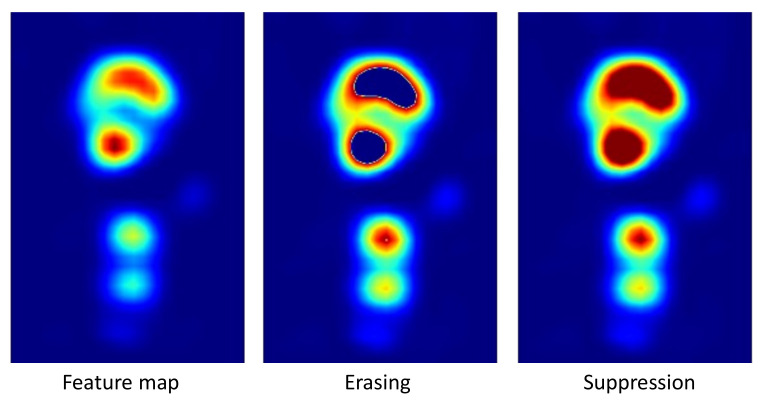
**Erasing and suppression**—Erasing sets to zero the areas above a certain threshold τ, while suppression sets those areas to τ. In this example, τ=0.6. The colors of the heatmaps are scaled based on their maximum and minimum values.

The ResNet-50 backbone is augmented with a Discriminative Region Suppression (DRS) module. DRS [48] works by suppressing feature map values with a maximum threshold that can be fixed or learned during the training phase. The result is that attention is spread to areas adjacent to the most active CAM regions instead of focusing on a few sparse discriminative object features, which is a common issue with standard CAM techniques [24,69,70]. The advantage of DRS is twofold: it has been designed for WSSS, thus can be applied to both single-instance and multi-instance data sets, and it works by suppressing and not erasing feature map values. Figure 2 demonstrates the difference between erasing and suppression on a given feature map. It can be noted that the fundamental difference between suppression and erasing is that suppression limits the maximum value of feature maps while erasing sets values higher than a threshold, i.e., the most important features, to zero. For this reason, erasing has been demonstrated to be detrimental in multi-class multi-instance scenarios [48] because it can make the network learn irrelevant features.

The DRS module is inserted in the ResNet-50 architecture after each skip-connection, only from conv3_x to conv5_x, since the previous layers conv1 and conv2_x, are frozen. We employ the learnable version of the DRS module, which has been demonstrated to obtain better localization performance than the fixed counterpart [48]. The insertion of the DRS module into the ResNet-50 architecture is evaluated in Section 4.2.

### 3.2. Class Activation Maps

Class Activation Mapping is an interpretability technique used to highlight the most discriminative class features inside images. Standard CAMs [37] are generated by performing, for each class, a weighted sum of the feature maps of the last convolutional layer. Given an input image *i* and a classification architecture, the actual CAM for a class *c*, indicated as $M_{i}^{c}$, is computed as follows:

(1) $$M_{i}^{c}=\sum_{k}w_{k}^{c}A_{k}$$

where $A_{k}$ is the *k*th feature map in the last convolutional layer, and $w_{k}^{c}$ is the weight associated with feature map *k* and class *c*.

Several CAMs variants have been proposed in the literature to address known limitations [71,72,73], yield better visualizations, and obtain finer localization. While some of them rely on the existing network structure [71,74,75,76,77,78], other techniques require the use of ad hoc architectures [73,79,80,81]. In this work, four state-of-the-art techniques will be analyzed for the generation of bounding boxes: CAM [37], Grad-CAM [74], Grad-CAM++ [75] and Smooth Grad-CAM++ [71]. For the implementation details, we refer the reader to the original works. A comparison of the localization abilities of CAMs on artworks has been presented in [17].

#### Percentile as a Standard for Thresholding

CAMs play a fundamental role in the generation of pseudo-GT bounding boxes. They are matrices of values between 0 and 1 representing each image pixel’s activation (or importance) to specific characteristics of each class. For the generation of bounding boxes from CAMs, a clear distinction between foreground and background areas must be made, and usually, the discrimination is performed by applying a fixed threshold [75,76,78]: values lower than the threshold are considered as background pixels and values higher or equal are assigned to the foreground. The works [17,82] studied the use of a fixed threshold and demonstrated that localization results strongly depend on the chosen value. The authors of [72] propose using Percentile as a Standard for Thresholding (PaS), which consists of using a threshold based on CAM values that better separates background and foreground areas. Their approach considers an image *i*, the normalized CAM $M_{i}^{c}$ associated with a class *c*, a percentile value p∈[0,100] and a fixed threshold $\theta_{loc}\in \left[0,1\right]$ with $p,\theta_{loc}\in R$. Hence, a localization threshold $\tau_{loc}$ is defined as:

(2) $$\tau_{loc}=\theta_{loc}\cdot {\mathrm{per}}_{p}\left(M_{i}^{c}\right)$$

where $\theta_{loc}$ is the fixed value usually employed when thresholding CAMs.

The percentile term (per${}_{p}$) considers the distribution of values inside each class activation map $M_{i}^{c}$. A percentile value *p* allows to obtain a single value ${\mathrm{per}}_{p}\left(M_{i}^{c}\right)$ that separates the (100−p)% highest values in $M_{i}^{c}$ from the p% lowest values and that, multiplied by a fixed parameter $\theta_{loc}$, constitutes the actual threshold $\tau_{loc}$. If p=100, ${\mathrm{per}}_{p}\left(M_{i}^{c}\right)=1$, and $\theta_{loc}=\tau_{loc}$, so PaS generalizes the fixed threshold approach. Class Activation Maps techniques and the introduction of PaS are studied in Section 4.2.

### 3.3. Object Detector

CAMs can accurately localize classes inside an image but lack the power to separate multiple instances correctly, especially when overlapping or very close. Furthermore, their quality has a strong dependence on the classification network. Hence, the need for a more robust object detector.

The final stage of the pipeline consists of Faster R-CNN [11], a state-of-the-art object detector commonly used alongside WSOD architectures. Faster R-CNN is a unified object detection network, composed of four subsequent modules: a Backbone Network for feature extraction, the RPN, the RoI Pooling Layer, and the classifier. The RPN module generates a set of anchor boxes at fixed positions and sizes and determines each anchor box’s objectness score. The RoI Pooling Layer, given a feature map and a set of proposals, extracts a pooled feature representation. Finally, the classifier associates a class label to each bounding box.

Faster R-CNN has been widely used in diverse fields, ranging from face recognition [83], to litter detection [84] and astrophysics [85]. In the literature, Faster R-CNN and other off-the-shelf object detectors have been used, in a weakly-supervised manner, to refine the object locations obtained from state-of-the-art WSOD architectures [9,30]. This research proposes to train Faster R-CNN with bounding boxes generated from CAMs and exploit the learned detection model to identify classes on non-natural artwork images.

The use of Faster R-CNN in the proposed pipeline and its robustness to noisiness and quality of pseudo-GT bounding boxes are analyzed in Section 4.2.

## 4. Evaluation

This section presents the data sets used for the task and the evaluation of each component. The evaluation aims at (1) understanding whether class activation maps combined with percentile thresholding are effective at localizing objects in different artworks’ data sets; (2) assessing if automatically generated pseudo-GT bounding boxes can be used as a replacement for manually annotated ground truth and (3) comparing the pipeline against state-of-the-art WSOD solutions. Both quantitative and qualitative analyses are presented.

### 4.1. Data Sets

To evaluate the proposed pipeline, two artworks’ data sets are used, namely ArtDL 2.0 and IconArt [16]. The two data sets were selected as representatives of non-natural images for the WSOD task, which was demonstrated to be quite different from WSOD on natural images [16,17,18]. They also provide diversity in the image quality and annotated classes.

#### 4.1.1. ArtDL 2.0

The ArtDL data set has been introduced in [14] with only image-level labels and extended with annotations for object detection in [17]. It contains 42,479 paintings representing the Iconclass [62] categories of 10 Christian Saints. The representation of Iconclasses in Christian art paintings uses specific symbols to identify the portrayed character, so the authors of [17] manually annotated 823 test images with bounding boxes for each Saint’s body and his/her related symbols for a total of 882 Saint-level and 2887 symbol-level annotations.

The ArtDL 2.0 data set adds bounding boxes that include both the Saints’ bodies and the associated symbols, because the latter are a proper part of the context that characterizes the iconography class and determines the detection.

**Figure 3:**
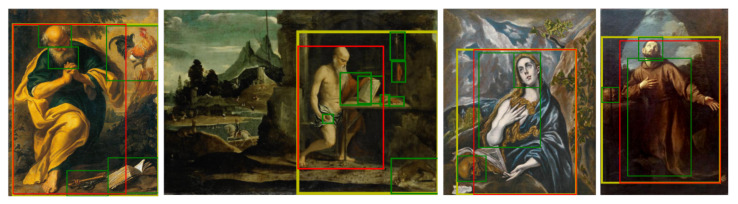
**ArtDL 2.0 bounding boxes**. The red bounding boxes contain the body of the saints and the green ones comprise individual iconography symbols associated with each Saint. The ArtDL 2.0 boxes (yellow) include both the Saint and the symbols.

Figure 3 shows some examples. The red and green bounding boxes are the original ArtDL annotations for the Saint and his/her iconographic symbols, while the yellow rectangle is the ArtDL 2.0 bounding box obtained by merging the red and green ones. As Figure 3 shows, some symbols may be inside a Saint-level box whereas others may be placed elsewhere in the image. Table 1 presents the statistics of the ArtDL 2.0 data set.

#### 4.1.2. IconArt

IconArt is a data set introduced in [16] to support the evaluation of WSOD techniques on artworks. This multi-class multi-label data set consists of seven classes portraying religious characters (e.g., Virgin Mary and Saint Sebastian) and non-religious subjects (e.g., ruins and nudity). No iconography symbols are associated with the classes. A validation set is randomly extracted from the training set following the procedure described in [16]. Table 2 reports the total number of images, the number of images used for detection, and the annotated bounding boxes of the IconArt data set.

### 4.2. Quantitative Analysis

This section starts by assessing the contribution of the DRS module to the classification performance. Then, it compares several CAM techniques and classification backbones for generating a pseudo-GT. Finally, it evaluates the proposed pipeline with regards to other state-of-the-art WSOD techniques.

#### 4.2.1. Classification

The evaluation of the classification performances has been conducted by comparing ResNet-50 enhanced with DRS and the architectures of the related works [14,18]. For the evaluation, a ResNet-50 model pre-trained on the ImageNet data set [66] has been used. The results of [14,18] have been replicated by applying the architectures and trained models provided by the authors. ResNet-50 with the inclusion of the DRS module has been trained with the same procedure of [14], both for ArtDL 2.0 and IconArt. The use of a fully-supervised image classification architecture on IconArt and Mi-max on ArtDL has not been documented previously.

Since the Mi-max architecture [16,18] is designed for end-to-end WSOD, the network’s output corresponds to bounding boxes instead of class confidence scores. To compute classification performance, the authors perform classification-by-detection. Thus, the image-level classification score for each class is defined as the highest detection score for that class.

Table 3 compares the classification results of the three architectures on the ArtDL 2.0 and IconArt data sets. The obtained results are comparable to those in the original works for all the replicated methods. ResNet-50 has consistent performance in the two data sets while ResNet-50 with DRS presents slightly lower precision and recall in ArtDL 2.0 and slightly better precision and worse recall in IconArt. These results are expected when employing attention modules because they focus on maximizing CAM localization rather than classification accuracy [86,87]. Mi-max shows much lower results, especially in ArtDL 2.0, due to the need to train the network from scratch and the difficulty in learning to identify the classes that present few examples and less discriminative features (e.g., Dominic, Anthony of Padua and Paul). The per-class AP results are presented in Table 4 and Table 5.

#### 4.2.2. Pseudo-GT Generation

Since Mi-max does not produce CAMs, only ResNet-50 and ResNet-50 with DRS are employed for the pseudo-GT generation step. The pseudo-GT bounding boxes have been evaluated on the validation set of ArtDL 2.0 for ResNet-50 with and without the DRS module. As for classification, results presented in [17] have been replicated as closely as possible.

The authors of [17] analyzed CAMs’ localization capabilities using a fixed threshold and found the optimal value to be 0.05. In this research, a grid search has been performed on threshold values $\theta_{loc}$ ranging from 0.05 to 1 with a step of 0.05 and on percentile values *p* ranging from 50 to 100 with a step of 5. Percentile values lower than 50 are not considered because they would always lead to a threshold close to 0, because most CAM values are 0 or very close to 0. The evaluation uses the Pascal VOC mAP metric. Since this evaluation assesses the ability of CAMs to generate pseudo-GT, the mAP value is computed only on GT classes. The aim is to have a pseudo-GT as similar as possible to human-annotated bounding boxes.

Table 6 summarizes the mAP results obtained on the ArtDL 2.0 validation set. Percentile-based thresholding yields better results than a fixed threshold for all CAM methods. ResNet-50 with DRS obtains better results on two methods (CAM and Grad-CAM++) and only when using fixed thresholds. Overall, the best combination is ResNet-50 without DRS and with CAM and percentile-based thresholding ($\theta_{loc}=0.15$ and p=95) with an mAP of 25.1%. This configuration is chosen to generate the pseudo-GT on the ArtDL 2.0 data set.

Table 6 shows that the threshold/percentile combinations vary across methods and backbones. For this reason and due to the differences between ArtDL 2.0 and IconArt (class types, number of bounding boxes per image, and image quality), an optimal threshold/percentile combination must be separately searched for the IconArt. Differently from ArtDL 2.0, IconArt has no validation set. The search strategy proposed in previous works [72,87,88] consists of two steps: first, a range of threshold and percentile values is set accordingly to ArtDL 2.0 results and standard suggested values. Then, a sample of 20 qualitative results is randomly drawn from the training set and analyzed to choose the best percentile/threshold values. The range considered for the percentile *p* is {85,90,95} and the range considered for the threshold $\theta_{loc}$ is {0.10,0.15,0.20,0.25}. The best qualitative results are obtained with ResNet-50 and the DRS module by setting $\theta_{loc}=0.20$ and p=90. On IconArt, the best results are obtained with ResNet-50+DRS because all the classes are represented by compact objects, so the region suppression feature delivers the expected benefits of a better focus. Conversely, ArtDL 2.0 classes may comprise multiple symbols not necessarily close to each other (see Figure 3).

To compare CAM techniques with a standard baseline, we adopted Selective Search [10], a region proposal method that computes a hierarchical grouping of superpixels based on color, texture, and size. Since Selective Search aims at a very high recall, the generated regions are classified by the architecture presented in Section 3.1 to keep only the most confident predictions as pseudo-GT (i.e., those predictions with a classification score ≥0.9). The obtained mAP is ≈0.03, much lower than the described CAM techniques.

An alternative solution has been presented in [89] to keep only tight discriminative boxes from Selective Search proposals. It consists of: (1) filtering all the boxes with a score lower than a threshold ($T_{score}$) and then applying Non-Maximum Suppression (NMS) to remove all the non-discriminative boxes; (2) discarding all the boxes that are entirely surrounded by a larger box; and (3) merging all the discriminative boxes with an IoU higher than a specified threshold ($T_{fusion}$). This technique can attain an mAP of 0.157 with $T_{score}=0.3$, NMS = 0.7 and $T_{fusion}=0.1$. This result is still ≈10% lower than CAM + PAS. For this reason, CAM + PAS has been used in this research for pseudo-GT mining.

#### 4.2.3. Weakly Supervised Object Detection

The final stage of the pipeline has been evaluated on the ArtDL 2.0 and IconArt test sets. For the classification stage, ResNet-50 (without DRS for ArtDL 2.0 and with DRS for IconArt) has been employed, while for the subsequent step the CAM and PaS have been selected. For ArtDL 2.0, the pseudo-GT bounding boxes have been generated with a threshold of 0.15 and percentile of 95, while for IconArt with 0.20 and 90.

For both data sets, a Faster R-CNN detector with a ResNet-50 backbone pre-trained on the Pascal VOC data set [90] is fine-tuned. The first two layers of the ResNet-50 backbone are frozen to exploit the transfer learning advantage that was confirmed to be essential for the classification stage. Bounding boxes for the evaluation are obtained by feeding test images directly to the trained object detector model, as presented in Figure 1. The first two stages (classification and pseudo-GT generation) are applied only to training images.

The proposed pipeline is compared against state-of-the-art WSOD architectures chosen based on their novelty and outstanding results on natural images: PCL [30], CASD [24] and UWSOD [36]. All methods have been replicated using the models provided by the respective authors trained with the recommended parameters, except those marked with * in Table 7 for which the results published by their authors are considered. Mi-max is included because it is the best performing state-of-the-art architecture on the IconArt data set. Bounding boxes generated with CAM and PaS have also been evaluated because they are a simple and commonly used technique and provide an interesting baseline.

Table 7 summarizes the results of the examined methods for both data sets. In ArtDL 2.0, all the end-to-end WSOD techniques present worse localization performance than CAM with PaS. In IconArt, the CAM + PaS and end-to-end methods yield similar results. While IconArt mAP values range from 3.2% of CAM + PaS to 16.6% of our method, for ArtDL 2.0 there is a vast difference between the 7.6% of UWSOD and the 41.5% of our method. This is justified by previous studies in the cultural heritage field, which demonstrates the importance of fine-tuning and transfer learning when working with non-natural images [14,15,52,68]. Most end-to-end architectures rely on frozen backbones, pre-trained on ImageNet or Pascal VOC, thus not allowing fine-tuning of the first stage of the network, which is fundamental for extracting features for the localization step. In addition, PCL, Mi-max, and CASD rely on external proposals from Selective Search [10] or from a Faster R-CNN RPN. Both alternatives present disadvantages: Selective Search was demonstrated in [18] to be unsuitable for artworks because proposals cover on average 50% of the GT bounding boxes, and fail with occluded or non-compact classes (e.g., those presented in Figure 3). Instead, RPN tends to cover objects similar to Pascal VOC’s classes but may not be relevant for non-natural images.

For ArtDL 2.0 it is interesting to note that PCL, a simpler WSOD technique, obtains better results and is more robust to training noise with regards to the more complex methods of CASD and UWSOD. Label noise and data set imbalance in ArtDL 2.0 also impact the performance of Mi-max. Table 7 also shows that CAMs performances vary much across data sets: a large drop is observed from ArtDL 2.0, which has few boxes per image to IconArt, which has many boxes per image, especially for some classes (angels, nudity).

Table 8 presents the per-class AP results on ArtDL 2.0, and Table 9 the per-class AP results on IconArt.

### 4.3. Qualitative Analysis

#### 4.3.1. Positive Examples

**Figure 4:**
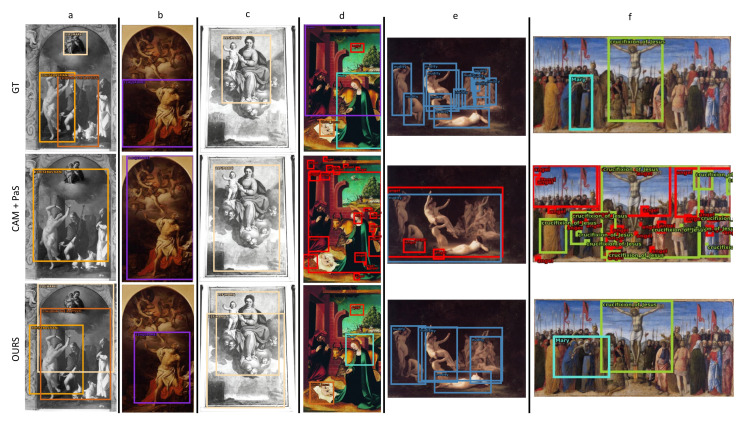
**Positive WSOD detections**—This figure presents positive examples on (**a**–**f**) six artworks from the ArtDL 2.0 and IconArt data sets. The first row contains the manually annotated ground truth, the middle row presents bounding boxes generated from CAM + PaS and the third row shows detections from the proposed WSOD method. Bounding boxes are color-coded by class for better visualization.

This section presents positive detection examples from the proposed method and compares them with the CAM + PaS baseline. Figure 4 shows three examples from the ArtDL 2.0 data set (a–c) and three examples from the IconArt data set (d–f). All six artworks present complex scenes depicting multiple characters of one or more classes.

In the ArtDL 2.0 data set, the bounding boxes produced by our method correctly locate the saints of interest and the most relevant symbols (e.g., Saint Sebastian’s arrows in Figure 4a, Saint Jerome’s writing material in Figure 4b and Baby Jesus in Figure 4c). Confusion is present when the Virgin Mary appears with other Saints due to imprecise pseudo-GT annotations. Compared to the baseline, our method better identifies the classes depicted in the images (e.g., in Figure 4a our pipeline recognizes all three classes of interest, while ResNet-50 recognizes only Saint Sebastian) while focusing on the most relevant areas rather than spreading on the entire image. Thus, even if Faster R-CNN is trained with knowledge from ResNet-50, the object detector can outperform the CAM baseline by discovering novel features.

Similar results are obtained for IconArt. The most relevant regions are identified correctly (e.g., in Figure 4d Mary’s upper part of the body, which is the most discriminative part, the child Jesus, and the very small angel). In Figure 4e, a crowded scene with nude figures is depicted. Our detector localizes and separates the majority of foreground figures into unique boxes, while enclosing the group in the background in one large box. Figure 4f shows another interesting example in which our method perfectly recognizes both Virgin Mary and the Crucifixion of Jesus even if the scene depicts many figures very similar to each other. The advantages discussed for the ArtDL 2.0 data set are even more evident in the IconArt data set, and the effect of the significant quantitative improvements presented in Table 9 can be noted in all the three artworks in Figure 4d–f.

#### 4.3.2. Negative Examples

**Figure 5:**
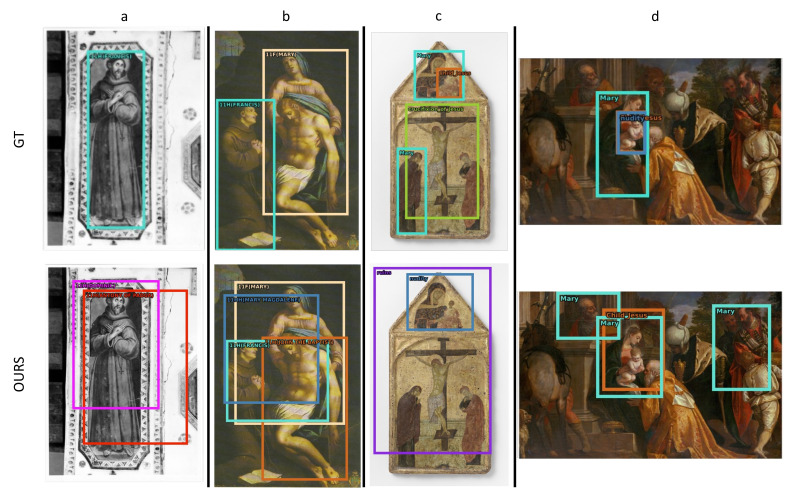
**Negative WSOD detections**—This figure presents negative examples on (**a**–**d**) four artworks from the ArtDL 2.0 and IconArt data sets. The first row contains the manually annotated ground truth and the second row shows detections from the proposed WSOD method. Bounding boxes are color-coded by class for better visualization.

Figure 5 presents two negative examples for the ArtDL 2.0 data set (a–b) and two for the IconArt data set (c–d). Our method can localize the depicted characters in the first two examples but fails at predicting the correct classes. In Figure 5a, the confusion depends on the similarity between Francis of Assisi, Dominic, and Anthony of Padua, which are the least represented saints in the data set and can be recognized by fewer distinctive symbols. In Figure 5b, the model makes some confusion between similar classes (Virgin Mary and Mary Magdalene) or figures that are often depicted together (e.g., John the Baptist and Jesus in “The Baptism of Jesus” scene). In Figure 5c, the model predictions are wrong. Virgin Mary and Child Jesus may be confused with nudity because in all the images the Child Jesus is associated with nudity, with the latter class being more recognizable. Figure 5d presents another common confusion between the Virgin Mary, Child Jesus, and Joseph or other male figures that are not annotated but frequently appear in the same scene.

**Figure 6:**
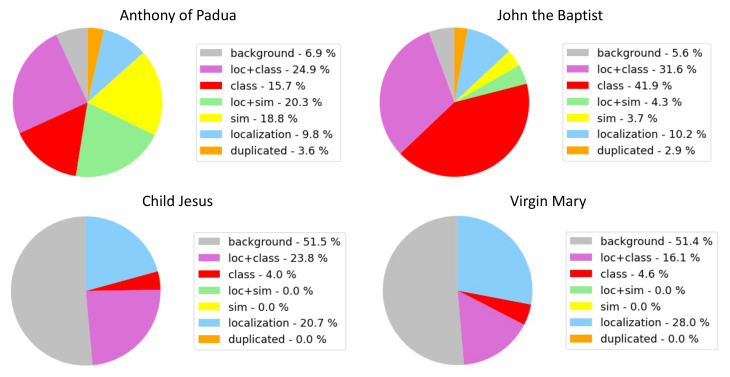
**FP error distributions**—The FP error analysis for two ArtDL 2.0 classes (Anthony of Padua and John the Baptist) and two IconArt classes (Child Jesus and Virgin Mary) shows a difference in error type distribution between the data sets.

False Positive (FP) examples can be analyzed by charting the error type distribution. For this evaluation, the ODIN framework [91] was used. Figure 6 shows the FP categorization for two ArtDL 2.0 classes (Anthony of Padua and John the Baptist) and two IconArt classes (Child Jesus and Virgin Mary). As shown in Figure 5a,b, the most common error related to Anthony of Padua and John the Baptist is confusion with other classes. For Anthony of Padua, confusion occurs with similar classes (39%) while for John the Baptist confusion happens mostly with other (not necessarily similar) classes. The IconArt data set presents completely different distributions: 50% of the errors are caused by confusion with background, i.e., the predictions are not intersecting with any GT box, while only 20–25% are due to misclassification and another 20–30% to poor localization. This difference is influenced by the images and scene characteristics of the two data sets, visible in Figure 4 and Figure 5.

## 5. Conclusions and Future Work

This paper presents a study on the effectiveness of a training pipeline for WSOD on non-natural (e.g., artwork) images. The proposed architecture is based on a combination of existing components. However, the results demonstrate that two-stage detection yields a simple yet effective solution on data sets characterized by label scarcity, uncommon classes, and less discriminative features.

The analysis has demonstrated that: (1) the introduction of the DRS module in the classification architecture is beneficial to delineating better CAM regions in data sets with multiple compact objects; (2) the use of PaS as a thresholding technique significantly improves localization for all the analyzed CAM techniques by adapting to varying image conditions (from black and white to very variable color palettes and confusing backgrounds); (3) CAM-based pseudo-GT generation on non-standard data sets can be used as a replacement of manual bounding box annotations, still generated bounding boxes present imperfections when compared to expert annotations; (4) faster R-CNN can successfully refine the localization knowledge derived from class-labels only, even on non-natural data sets; and (5) end-to-end WSOD architectures are ineffective on the considered artworks’ data sets.

Future work will focus on re-using the previously trained ResNet-50 classification model as the backbone for Faster R-CNN. This would allow exploiting the already learned weights and most discriminative features while also making the training of Faster R-CNN lighter and faster. Novel CAM-based [92] or box-based [93,94] Weakly Supervised Instance Segmentation approaches will also be studied to obtain more precise localization. Still, these methods would require an extensive manual annotation of test images. Finally, an interesting future application consists in addressing the captions’ generation problem [95] by integrating the trained object detector (e.g., Faster R-CNN) in a caption generation network [96,97], possibly exploiting hierarchical relations in paintings [98].

## Figures and Tables

**Table 1:** **The ArtDL 2.0 data set**—The number of images is shown per class and per task (classification and object detection). For object detection, the number of annotated bounding boxes is reported too.

	Set	Virgin Mary	Antony of Padua	Dominic	Francis of Assisi	Jerome	John the Baptist	Paul	Peter	Sebastian	Mary Magd.	Total
Classification images	Train	9515	115	234	784	939	943	419	949	448	727	15073
	Val.	1189	14	30	98	117	97	52	118	56	90	1861
	Test	1189	14	29	98	118	99	52	119	56	90	1864
Object detection images	Val.	1063	23	30	98	101	101	40	86	54	85	1625
	Test	283	26	29	85	99	81	34	84	47	68	823
Annotated bounding boxes	Val.	1076	23	30	98	102	101	40	87	55	85	1697
	Test	283	26	29	85	99	81	34	84	47	68	836

**Table 2:** **The IconArt data set**—The number of images is shown per class and per task (classification and object detection). For object detection, the number of annotated bounding boxes is reported too.

	Set	Angel	Child Jesus	Crucifixion	Mary	Nudity	Ruins	Saint Sebastian	*None*	Total
Classification images	Train	600	755	86	1065	956	234	75	947	2978
	Test	627	750	107	1086	1007	264	82	924	2977
Object detection images	Test	261	313	107	446	403	114	82	623	1480
Annotated bounding boxes	Test	1043	320	109	502	759	194	82	*N/A*	3009

**Table 3:** **Classification performance**—This table presents the classification results on the ArtDL 2.0 and IconArt data sets. Reported results are macro-averaged. ResNet-50 and ResNet-50 with DRS show similar and consistent results in the two data sets while Mi-max is the architecture obtaining the worst classification results. The best-performing method for each data set is highlighted in bold.

Method	ArtDL 2.0				IconArt			
	Precision	Recall	F1	AP	Precision	Recall	F1	AP
ResNet-50 [14]	0.727	0.698	**0.691**	**0.716**	0.715	0.679	0.642	0.725
ResNet-50 + DRS	0.658	0.658	0.649	0.701	0.717	0.619	**0.656**	**0.731**
Mi-max [18]	0.040	0.850	0.090	0.176	0.240	0.970	0.360	0.540

**Table 4:** **Per-class classification performance on ArtDL 2.0**—This table presents the per-class AP results on the ArtDL 2.0 data set. The best-performing method for each class is highlighted in bold.

Method	Virgin Mary	Antony of Padua	Dominic	Francis of Assisi	Jerome	John the Baptist	Paul	Peter	Sebastian	Mary Magd.
ResNet-50	**0.973**	0.548	0.498	0.746	**0.784**	**0.805**	**0.469**	**0.733**	**0.822**	**0.781**
ResNet-50 + DRS	0.959	**0.737**	**0.575**	**0.782**	0.781	0.707	0.345	0.663	0.790	0.675
Mi-max	0.768	0.016	0.017	0.059	0.266	0.208	0.049	0.074	0.390	0.189

**Table 5:** **Per-class classification performance on IconArt**—This table presents the per-class AP results on the IconArt data set. The best-performing method for each class is highlighted in bold.

Method	Angel	Child Jesus	Crucifixion	Mary	Nudity	Ruins	Saint Sebastian
ResNet-50	**0.739**	**0.848**	0.794	**0.888**	**0.821**	0.764	0.219
ResNet-50 + DRS	0.702	0.841	**0.833**	0.883	0.818	**0.789**	**0.250**
Mi-max	0.548	0.547	0.765	0.694	0.651	0.412	0.138

**Table 6:** **Mean Average Precision of pseudo-GT (ArtDL 2.0)**—The mAP is evaluated considering the CAM of the GT classes on the validation set. The use of percentile always yields better results than a fixed threshold and the best architecture is the configuration with ResNet-50, CAM, a threshold of 0.15, and the 95th percentile. The best-performing method is highlighted in bold.

Method	DRS	Fixed Threshold		PaS		
		Threshold	mAP	Threshold	Percentile	mAP
CAM [37]	✗	0.05	0.184	0.15	95	**0.251**
	✓	0.10	0.208	0.35	95	0.213
Grad-CAM [74]	✗	0.05	0.174	0.55	90	0.231
	✓	0.10	0.150	1.00	75	0.180
Grad-CAM++ [75]	✗	0.05	0.158	0.15	95	0.229
	✓	0.10	0.166	1.00	80	0.215
Smooth Grad-CAM++ [71]	✗	0.05	0.167	0.65	85	0.229
	✓	0.10	0.152	1.00	80	0.183

**Table 7:** **WSOD Performance**—Detection capabilities are evaluated on the test set using the Pascal VOC mAP metric. The results marked with * are taken from the original works. The best-performing architecture for each data set is highlighted in bold.

Architecture	ArtDL 2.0	IconArt
PCL [30]	0.248	0.059 *
CASD [24]	0.135	0.045
UWSOD [36]	0.076	0.062
Mi-max [18]	0.082	0.145 *
CAM + PaS	0.403	0.032
Ours	**0.415**	**0.166**

**Table 8:** **Per-class WSOD performance on ArtDL 2.0**—Detection capabilities are evaluated on the ArtDL 2.0 test set for each class using the AP metric. The best-performing architecture for each class is highlighted in bold.

Architecture	Virgin Mary	Antony of Padua	Dominic	Francis of Assisi	Jerome	John the Baptist	PAUL	Peter	Sebastian	Mary Magd.
PCL	0.478	0.024	0.005	0.122	0.476	0.204	0.059	0.191	0.370	0.554
CASD	0.301	0.011	0.035	0.059	0.344	0.057	0.010	0.112	0.072	0.351
UWSOD	0.018	0.063	0.033	0.022	0.022	0.034	0.018	0.019	0.023	0.014
Mi-max	0.142	0.016	0.000	0.000	0.128	0.112	0.024	0.040	0.219	0.136
CAM + PaS	0.242	**0.341**	0.254	0.282	**0.604**	0.308	**0.268**	**0.613**	0.418	**0.697**
Ours	**0.490**	0.230	**0.322**	**0.294**	0.551	**0.468**	0.245	0.540	**0.446**	0.556

**Table 9:** **Per-class WSOD performance on IconArt**—Detection capabilities are evaluated on the IconArt test set for each class using the AP metric. The best-performing architecture for each class is highlighted in bold.

Architecture	Angel	Child Jesus	Crucifixion	Mary	Nudity	Ruins	Saint Sebastian
PCL	0.029	0.003	0.010	**0.263**	0.023	0.014	0.072
CASD	0.002	0.000	0.200	0.049	0.014	0.023	0.028
UWSOD	**0.089**	0.000	0.020	0.016	0.003	0.076	0.112
Mi-max	0.043	**0.067**	0.357	0.156	0.240	**0.152**	0.001
CAM + PaS	0.010	0.002	0.076	0.009	0.028	0.052	0.046
Ours	0.009	0.017	**0.589**	0.019	**0.243**	0.061	**0.221**

## Data Availability

Publicly available datasets were analyzed in this study. The data can be found on http://www.artdl.org (accessed on 1 July 2022) and https://wsoda.telecom-paristech.fr/downloads/dataset/ (accessed on 1 July 2022).
